# Implications of siRNA Therapy in Bone Health: Silencing Communicates

**DOI:** 10.3390/biomedicines12010090

**Published:** 2024-01-01

**Authors:** Puneetpal Singh, Monica Singh, Baani Singh, Kirti Sharma, Nitin Kumar, Deepinder Singh, Harpal Singh Klair, Sarabjit Mastana

**Affiliations:** 1 Department of Human Genetics, Punjabi University, Patiala 147002, Punjab, India; singhmonica2017@gmail.com (M.S.); baani_rs22@pbi.ac.in (B.S.); kirti_rs22@pbi.ac.in (K.S.); nitin_rs19@pbi.ac.in (N.K.); 2 Vardhman Mahavir Health Care, Urban Estate, Ph-II, Patiala 147002, Punjab, India; 3 Klair Orthopaedic Centre, Punjabi Bagh, Patiala 147001, Punjab, India; 4 Human Genomics Laboratory, School of Sport, Exercise and Health Sciences, Loughborough University, Loughborough LE11 3TU, UK

**Keywords:** RNAi, siRNA therapy, bone disorders, siRNA delivery

## Abstract

The global statistics of bone disorders, skeletal defects, and fractures are frightening. Several therapeutic strategies are being used to fix them; however, RNAi-based siRNA therapy is starting to prove to be a promising approach for the prevention of bone disorders because of its advanced capabilities to deliver siRNA or siRNA drug conjugate to the target tissue. Despite its ‘bench-to-bedside’ usefulness and approval by food and drug administration for five siRNA-based therapeutic medicines: Patisiran, Vutrisiran, Inclisiran, Lumasiran, and Givosiran, its use for the other diseases still remains to be resolved. By correcting the complications and complexities involved in siRNA delivery for its sustained release, better absorption, and toxicity-free activity, siRNA therapy can be harnessed as an experimental tool for the prevention of complex and undruggable diseases with a personalized medicine approach. The present review summarizes the findings of notable research to address the implications of siRNA in bone health for the restoration of bone mass, recovery of bone loss, and recuperation of bone fractures.

## 1. Introduction

The global figures of individuals suffering from musculoskeletal conditions, bone defects, and bone fractures are frightening and impinge harshly on health statistics [1]. Data from 204 countries have exposed that 1.71 billion individuals of all ages are suffering from musculoskeletal abnormalities, contributing substantially to years lived with disability (YLD) and disability-adjusted life years (DALY) [2]. Any episode of bone degeneration or deformity, infection or inflammation, tumor or trauma, compromised bone mass or bone density, frailty, or fracture invokes endogenous repair mechanisms, including bone remodeling, revascularization, callus formation, mineralization, hormonal regulation, and stem cell differentiation. These mechanisms are supported by medical interventions and regenerative therapies to ensure effective restoration of bone structure, recovery of strength, and recuperation from bone loss. 

Processes of bone repair, regeneration, and healing are vital for the maintenance of skeletal integrity, mobility, and overall health. Despite these mechanisms remaining in place, many times bone repair and regeneration fail to match the degree of destruction and damage to bone tissue due to several reasons, including impaired angiogenesis, persistent generation of reactive oxygen species (ROS), endothelial dysfunction, dysregulated neutrophils, uncontrollable inflammation, and stem cell dysfunction [3,4,5,6]. However, the primary reason for this perturbed homeostasis of bone repair is the intricate interactions and cross-talks of signaling pathways that induce an unmanageable auto-immune response [5]. The alarming figures of global musculoskeletal conditions and bone fractures have engrossed the attention of scientists and scholars to explore different interventional strategies for bone repair, regeneration, and healing [1].

The scientific venture to repair bone defects, deformities, and fractures with bone grafts, growth-promoting factors, pharmacological interventions, tissue engineering, infusions of scaffolds, and mesenchymal and induced pluripotent stem cells is challenging as these are considered exogenous invaders and invoke an auto-immune response. In the last few years, RNA interference (RNAi)-based regulation of gene function has had an astounding impact on reforming the therapeutic potential of several diseases. RNAi is an evolutionarily maintained biological defense mechanism that is triggered in response to double-stranded RNAs (dsRNAs) and silences gene function by degrading mRNA in a sequence-specific manner [7]. Consequently, scientists have realized that particular sequences of dsRNAs can be generated artificially to silence the gene that causes a particular disease. Two types of dsRNA, i.e., small interference RNA (siRNA) and micro RNA (miRNA), are important components proven to be used for gene silencing through the RNAi mechanism.

Several exogenous and endogenous triggers, like double-stranded (ds) DNA of the viral genome, aberrant transcripts formed from transposons and miRNA primary transcripts of pathogens, and the process of post-transcriptional gene silencing (PTGS) mediated by siRNA, occur through RNAi signaling [8]. Initially, dsRNA hairpin loops or short hairpin RNAs (shRNAs) are recognized and cleaved by the DICER enzyme. It is an evolutionarily conserved helicase with an endoribonuclease motif that belongs to the ribonuclease III family. The action of DICER on shRNA results in its degradation to form double-stranded siRNA(ds-siRNA), comprising 21–22 nucleotides that contain overhangs at the 3′end and free phosphate groups at the 5′ end [9]. The siRNA now consists of two strands: a passenger strand (the sense strand) and a guide strand (the anti-sense strand). This ds-siRNA is then incorporated into the RNA-induced silencing complex (RISC), also known as the RNA-induced transcriptional silencing complex (RITSC). RISC is a multi-ribonucleoprotein complex composed of four domains: the exonuclease domain (EXO), the endonuclease domain (ENDO), the domain containing homology-searching activity related to *E. coli* (RecA), and the helicase domain. The EXO, ENDO, and RecA are dsRNA-binding domains with nucleolytic activity, and the helicase domain, formed by Argonaute protein (AGO), is responsible for the ATP-dependent RNA helicase activity of RISC, which leads to the unwinding of ds-siRNA into a single strand by degradation of its passenger strand. The guide strand of siRNA leads the RISC to the target mRNA, subsequently causing its cleavage from the center at a single site by the ENDO domain of the RISC, whereby Argonaute 2 (AGO2) breaks the phosphodiester bond, resulting in the target gene silencing [10].

For the first time, its therapeutic importance was realized when synthetic siRNAs were transfected in an experimental mouse model of the hepatitis C virus, which silenced its expression [11]. In line with this, siRNAs were directed against the mRNA of the Fas cell surface death receptor (FAS gene) in an autoimmune hepatitis mouse model, which silenced Fas-expression and consequently protected mice from liver failure and fibrosis [12]. Five siRNA-based therapeutic medicines, i.e., Patisiran (ONPATTRO) and Vutrisiran (AMVUTTRA), for the treatment of heredity transthyretin-induced amyloidosis with polyneuropathy (hATTR), Inclisiran (LEQVIO), Lumasiran (OXLUMO), and Givosiran (GIVLAARI), have been approved by the United States Food and Drug Administration (USFDA)for the treatment of hypercholesterolemia, primary hyperoxaluria type 1, and hepatic porphyria, respectively [13,14,15,16]. Nonetheless, their effective delivery at the site of the problem is still challenging. Though it has been tried and tested in rats/mice, rabbits, and horses for several disorders, the implications of siRNA-based therapeutics for bone disorders, deformities, and fractures in humans are still in their infancy.

With an alarming increase in epidemiological parameters and morbidityassociated with bone diseases, RNAi-based therapy using siRNA molecules is a promising strategy to ameliorate bone disorders. The current narrative review highlights the eminent research findings to acknowledge the indispensable role played by siRNA in bone health for bone mass rehabilitation, bone loss recuperation, and bone fracture healing, along with a variety of delivery systems that aid in siRNA delivery to the bone and their consequences.

## 2. Methodology

A literature survey was carried out by browsing the biomedical search engines, i.e., PubMed, EMBASE, Google Scholar, and Cochrane Library for original articles, review articles, meta-analyses, and systematic reviews with the keywords “siRNA in bone health”, “siRNA therapeutics in bone disorders”, “siRNA and IVDD”, “siRNA and rheumatoid arthritis, “siRNA and bone fractures”, “siRNA and osteoarthritis” and “siRNA and osteoporosis” for siRNA-based therapy for bone defects, and keywords including “siRNA delivery systems in bone”, “siRNA-nanoparticle conjugates and bone”, and “siRNA nanocarriers for bone diseases” were used for delivery methods to deliver siRNA to the bone. Moreover, references to reviews and meta-analyses were also screened to select the relevant research publications. Only those articles were selected that were published in the English language. All the research papers selected for the review were published in reputed journals. A total of 330 articles were collected from January 2013 to November 2023, out of which 126 research papers were selected, from which the information was extracted and interpreted for this narrative review.

## 3. Therapeutic Interventions of siRNA in Major Bone Disorders

The utility of siRNA therapy has been examined in several disorders, with success in many and failure in some reports [17,18,19,20]. However, the management of bone disorders with siRNA therapy in humans and their clinical relevance from ‘bench to bedside’ is blurry because most of the studies have been carried out on mouse, rabbit, and horse models [17,18,19,21,22]. Some hitches and hiccups in their design and delivery have been noticed, which have guided the scientific fraternity to develop novel methods for their better use and utility with more efficiency. The usefulness of siRNA-based management of bone disorders in humans, such as osteoporosis, osteoarthritis, rheumatoid arthritis, intervertebral disc disorder, and fractures, has been compiled and discussed to understand the possibilities of filling gaps in the knowledge of siRNA therapeutics in these disorders for their future perspectives.

### 3.1. Small Interfering RNA Therapy for Osteoporosis 

Osteoporosis is a systemic skeletal disease manifested as weak bones due to low bone mass and the degraded microstructure of the bone tissue. The epidemiological statistics of osteoporosis are frightening, as every third second there is an osteoporosis-related fracture occurring somewhere in the world. More than 8.9 million fractures are reported annually, and their impact is so severe that an individual comes to know that he/she is suffering from osteoporosis after his/her first fall/fracture [23]. In the face of the advancing challenge to prevent and manage osteoporosis, siRNA therapy has started showing effective results. A few years ago, siRNA was designed to target the inhibition of receptor activator of nuclear factor kappa B (NF-kβ; RANK) to block osteoclastogenesis for the prevention of osteoporosis. This siRNA was delivered by a novel method of delivery known as biocompatible mesoporous bioactive glass (MBG) to the osteoclast cells. The efficiency of siRNA-RANK-MBG to inhibit osteoclastogenesis was observed to be 70 percent, along with significant attenuation of the gene expression of C-Fos, Cathepsin-k (CTSK), Nuclear factor of activated T cells cytoplasmic 1 (NFATc1), and Tartrate-resistant acid phosphatase (TRAP) genes [24]. Another study showed siRNA therapy against heat shock protein 90 (HSP90), which is a significant player in glucocorticoid-induced osteoporosis (GIOP) [25]. siRNA conjugated with 17-Demethoxy-17-allyaminogeldassmyin (17-AAG), which is an inhibitor of HSP90, was used, which significantly reduced the transcriptional output of glucocorticoid signaling, whereas overexpression of HSP90α and HSP90β augmented glucocorticoid expression in calvarial osteoblasts. This finding suggests that bone formation can be preserved by targeting HSP90 with siRNA-17-AAG in GIOP. It is known that Casein kinase 2 interacting protein (CKIP-1) is a negative regulator of bone formation, irrespective of bone resorption. CKIP-1-siRNA was designed, and its effect on osteogenic differentiation, bone mineralization, and bone mass parameters was observed for osteoporosis intervention [26]. The results were encouraging, as the silencing of CKIP-1 mRNA expression increased bone formation significantly without inducing any autoimmune response. Similarly, the sclerostin (SOST) gene, which downregulates Wingless-related integration site (Wnt) signaling and suppresses osteoblast differentiation, was targeted with mesoporous silica nanoparticles (MSNs) coated with SOST-siRNA. It increased ostegenic marker expression and, hence, showed positive results for the prevention of bone mass loss in osteoporosis [27]. The CTSK gene is implicated in bone degradation because of its predominant role in enhancing osteoclastogenesis. To silence its expression and preserve bone mass in osteoporosis, siRNA was designed with an ultrasound-responsive nanodrop, encapsulated with CTSK, and embedded with alendronate (AL) for bone osteoclasts (CTSKsiRNA-ND-AL). This therapy suppressed osteoclastogenesis significantly, highlighting a novel strategy of ultrasound-responsive targeted nanodroplet processes for both gene and drug delivery [28]. Crk proto-oncogene (CrkII), an adaptor protein, plays a key role in cytoskeletal reorganization, phagocytotic cup formation, mitogenesis, osteoclast differentiation, and osteoblast differentiation. CrkII expression promotes c-jun-N-terminal kinase (JNK) phosphorylation and slows down osteoblast differentiation. Moreover, its overexpression reduces bone mass; hence, it is considered a negative regulator of bone remodeling. CrkII siRNA was encapsulated in a bone-targeting peptide (AspSerSer)^6^-liposome to target receptor activator of nuclear factor kappa-B ligand (RANKL) signaling to improve bone mass in vitro [29]. Microcomputed tomography revealed that RANKL-induced bone loss was recovered by systemic administration of AspSerSer^6^-liposome-CrkII siRNA therapy, which suggested it to be an efficient method for the treatment of bone disorders with bone-specific delivery of siRNA. In similar lines of siRNA therapy, ultrasound-responsive nanobubbles (NB) were used as controlled-release carriers of siRNA CTSK and cerium oxide nanoparticles (CeNPs) to prevent osteoporosis and improve bone mass by downregulating CTSK-induced osteoclastogenesis and upregulating CeNP-induced osteogenesis [30]. The results have suggested that delivery of CTSK-CeNP-siRNA by ultrasound-responsive NBs can be an effective treatment for osteoporosis and associated bone fractures.

### 3.2. Small Interfering RNA Therapy for Osteoarthritis

Knee osteoarthritis is a painful joint disorder that is irreversible, causing deformity by degrading articular cartilage, leading to pain and joint dysfunction. It has been revealed that infusion of adipose-derived stem cells (ADSCs) into the knee joint cavity may improve the repair and healing process in knee osteoarthritis. Gene expression profiling of ADSCs in synovial fluid has suggested that they can activate several genes. To understand the role of gene expression for cell viability, siRNA was designed to target FOS like 1, the AP-1 Transcription Factor subunit (FOSL1), and was infused in ADSCs, which showed reduced cell viability, suggesting that FOSL1 is responsible for ADSCs survival in synovial fluid in knee osteoarthritis [31]. This study recommended that for better outcomes in knee osteoarthritis, cultured ADSC-induced therapy should be supplemented with upregulation of FOSL1 expression. High mobility group box chromosomal protein 1 (HMGB-1) plays a significant role in oxidation, inflammation, and apoptosis within chondrocytes, contributing to the development of osteoarthritis. HMGB siRNA was designed and delivered to chondrocytes of knee osteoarthritic patients, which revealed that silencing HMGB-1 mRNA expression significantly attenuated the inflammatory signaling by mediating the effects of matrix metallopeptidase 13 (MMP13), Collagenase, Interleukin-6 (IL-6), and Collagen type II alpha 1 (COL2A1) genes [32]. Chondrocytes are generally inaccessible because of the dense extracellular matrix, so delivery of therapeutic siRNA insitu to articular cartilage is challenging. Self-assembly peptide nanoparticles (NP) can deliver up to a depth of 700 µm. Therefore, siRNA-NP targeted against nuclear factor kappa B (NF-kB)p^65^ was designed, which significantly reduced chondrocyte apoptosis and improved cartilage homeostasis to prevent cartilage deterioration in osteoarthritis [33]. 

**Figure 1:**
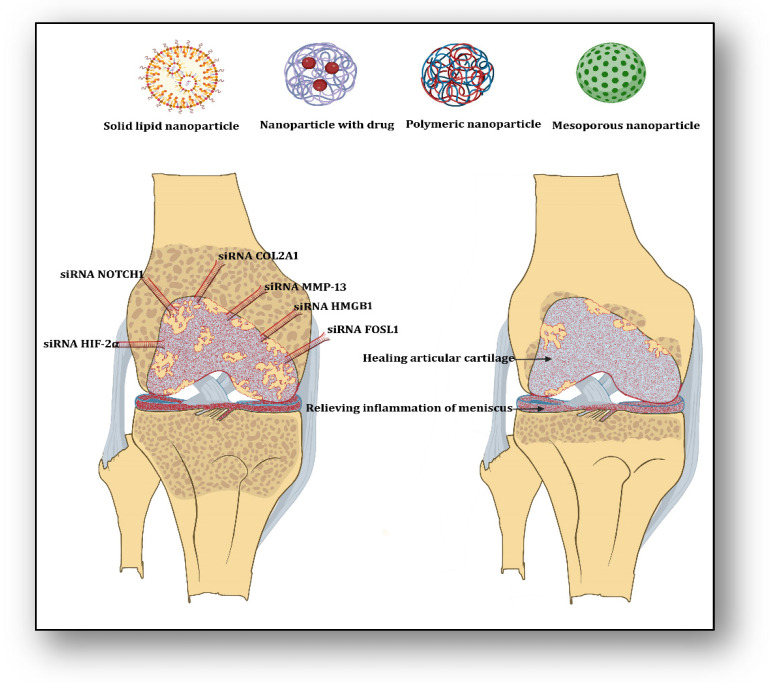
Showing several proven siRNAs and their delivery carriers for the gene-silencing-mediated treatment of Knee osteoarthritis. Created with Adobe Photoshop.

To understand the effect of nitric oxide (NO) in inflammatory cells, photothermal-triggered NO nanogenerators, NO-Hb@siRNA@PLGA-PEG (NHsPP), were designed for the prevention of osteoarthritis. In this case, hemoglobin (Hb) acted as a NO carrier, which absorbed infrared light (650 nm) and converted it into heat to generate NO. This assembly was loaded with Notch 1-siRNA, which exerted therapeutic effects by inhibiting the synthesis of proinflammatory cytokines and macrophages [34]. This therapy has suggested a novel non-invasive photothermal nanoparticle-based NO-releasing technology to manage inflammatory signaling therapy in osteoarthritis. Anti-inflammatory and immunosuppressive Interleukin 37 (IL-37) plays a role in converting proinflammatory macrophage 1 (M1) to anti-inflammatory M2 during inflammation. To have beneficial effects of IL-37, siRNA-IL-1R8 and MCC-950 were designed and delivered, which exhibited that IL-37 inhibited the expression of Nucleotide-binding domain, leucine-rich, pyrin domain-containing-3 (NLRP3) inflammasome-derived release of proinflammatory cytokines IL-18 and IL-1β along with upregulation of IL-1R8 expression [35]. This inference has suggested the utility and usefulness of siRNA IL-1R8 to suppress inflammasome-based inflammatory cascades in bone disorders. One may imagine that the application of several proven siRNAs may reduce the inflammatory episodes, reverse articular cartilage degradation, and relieve pain in knee osteoarthritis (Figure 1). Lately, a pH-responsive metal organic framework (MIL-101-NH_2_) was developed for the delivery of curcumin and targeted delivery of hypoxia-inducible factor 2 subunit alpha (HIF-2α). MIL-101-NH_2_ helped siRNA HIF-2α degradation from nucleases by lisosomal escape. This MIL-101-NH_2_ framework released the siRNA payload in an acidic environment and downregulated HIF-2α expression, leading to blocking inflammatory signaling and cartilage degradation in osteoarthritis [36].

### 3.3. Small Interfering RNA Therapy for Rheumatoid Arthritis

Rheumatoid arthritis (RA) is a painful and disabling disease of the joints and other body parts due to the overt expression of autoimmune and inflammatory signaling leading to bone erosion and joint deformity. Pharmacological strategies encompassing non-steroidal anti-inflammatory drugs (NSAIDS) to disease-modifying anti-rheumatic drugs (DMARDs), inhibitors of cytokines, chemokines, T cells, and receptor antagonists of interleukins, along with immunomodulatory interventions, are in use to alleviate the bone degradation and painful manifestations in RA. siRNA therapy to block those molecular signaling pathways that encourage autoimmune response and inflammation is the concept of a new era of molecular medicine to curb the processes, pathways, and problems of RA. In this direction, some of the studies have tried to target the mRNA expression of those genes that actively participate in different phases of RA pathology. For instance, protein arginine methyl transferase 5 (PRMT5) plays a significant role in several cellular processes, including cell proliferation, transcriptional regulation, inflammation, the assembly of small nuclear ribonucleoproteins, and the invasion of fibroblast-like synoviocytes (FLSc). siRNA was designed to target the PRMT5 gene with its inhibitor EPZ015666 and delivered to the synovium of an inflamed joint [37]. The results showed that silencing the PRMT5 gene by EPZ015666 significantly reduced the production of proinflammatory cytokines IL-6 and IL-8 in FLSc and slowed down the invasion and proliferation of FLSc in inflamed synovium. This study has suggested that targeting the PRMT5 gene is a better option to reduce inflammation and invasion of RA FLSc by downregulating the NF-kB and AKT pathways. Similarly, the 6-phosphofructo-2-kinase/fructose-2,6-bisphosphatase 3 (PFKFB3) enzyme supports invasion of FLSc and glycolytic metabolism-induced inflammation in RA. siRNA with the PFKFB3 inhibitor PFK15 was designed and delivered to the synovial fluid of RA patients [38]. This siRNA-based inhibition of PFKFB3 attenuated the expression of IL-8, IL-6, Chemokine (C-C motif) ligand 2 (CCL-2) and Chemokine interferon-γ inducible protein 10kDa (CXCL-10) along with a significant reduction in migration and invasion of FLSc. The inference of this study has suggested that PFKFB3 expression and FLSc invasion can be blocked to prevent synovial inflammation and degradation of joints in RA. FLSc-induced inflammatory signaling and its invasion into the intracellular matrix are crucial to RA pathology. To target RA FLSc, siRNA was designed with Ribonucleotide reductase M2 (RRM2) and loaded onto a peptide-conjugated liposome-polycation-DNA (LPD) complex [39]. RRM2 negatively regulates osteogenesis, repairs DNA damage, and inhibits cellular apoptosis. Silencing RRM2 significantly augmented apoptosis and reduced the proliferation of RA FLSc. Furthermore, the production of proinflammatory cytokines such as tumor necrosis factor alpha (TNF-α) and IL-6 was markedly reduced, leading to better management of RA patients. Bone morphogenetic protein 3 (BMP3) reduces inflammation signaling in response to FLS in RA, but its reduced expression in synovium encourages invasion of FLSc in the pannus (irregular growth in the joint), contributing to the RA pathology. Its importance was understood by designing siRNA against BMP3 in RA patients, which revealed that inhibition of BMP expression augments the synthesis of proinflammatory cytokines such as IL-6, IL-1β, TNF-α, and IL-17A and chemokines such as CCL-2 and CCL-3, along with MMP-3 and MMP-9 [40]. Blocking the expression of BMP increases the migration of FLSc stimulated by TNF-α. Results of this study have indicated that BMP3 is responsible for suppressing migration and invasion of FLSc in synovial fluid in RA patients. 

Myeloid Cell Leukemia 1 (MCL1) is an anti-apoptotic oncoprotein that regulates hematopoetic cell survival and differentiation. siRNA against MCL1 triggers macrophage apoptosis, which can relieve inflammation and joint pain [41]. Composite microspheres (MPs) were loaded with Chitosan (CS) and Hyaluronic acid (HA) nanoparticles. This siRNA, HA-CS-NP (HCNP), was loaded into poly D,L-lactide-co-glycolide (PLGA), and poly cyclohexane 1,4-diyl acetone dimethyleneketal (PCAK) composite microspheres. This novel method of NPs-in-MPs (NiMPs) helped in the sustained release of NPs and protected siRNA from nucleases. The results of this study have suggested that using NiMPshas a better technological advantage and more pharmacodynamic effect than NPs and HCNPs, making it a novel strategy for delivering siRNA for RA therapy. The role of TNF-α and Heterogenous nuclear ribonucleoprotein L-related immunoregulatory long non-coding RNA (THRIL) for the invasion and migration of FLSc in synovium, cartilage, and bone was investigated [42]. siRNA THRIL and lentivirus overexpressing it were used to silence as well as overexpress THRIL in different patients. Blocking THRIL expression increased the invasion and proliferation of FLSc, whereas overexpression of THRIL reduced MMP-13 expression in response to an IL-1β-induced stimulus. THRIL knock-down significantly increased the production of IL-1β-induced signaling of MMP-1, MMP-3, and MMP-13 expression. The results of this study recommend that long non-coding (lnc) RNA-based THRIL targeting is a better way to manage RA. During the repair and regeneration of an inflamed joint, class 3 semaphorins, which are potent mediators of angiogenesis, are required, but they are generally reduced in the synovial fluid of RA patients. The expression of class 3 semaphorins is mediated by many transcription factors, but homeobox protein-5 (HOXA5) is the chief regulator for the expression of class 3 semaphorins by FLS and endothelial cells. siRNA targeting mRNA of the HOXA5 gene significantly attenuated the expression of class 3 semaphorins in FLS and human umbilical vein endothelial cells (HUVEC) within the synovium of RA patients [43]. Silencing of HOXA5 also increased the invasion and migration of FLSc, hence making it a novel mechanism for regulating hyperplasia of synovium by controlling class 3 semaphorins. Lately, a study has suggested reprograming living neutrophils for cytopharmaceuticalsloaded with TNF-α siRNA to inhibit TNF-α production by macrophages in inflamed synovium [44]. This TNF-α siRNA/neutrophils cytopharmaceutical can easily invade synovium and submit TNF-α siRNA to macrophages, which decreases TNF-α-induced inflammation and blocks neutrophil inflammatory signaling, leading to lessening joint inflammation and enhancing cartilage repair.

### 3.4. Small Interfering RNA Therapy for Intervertebral Degenerative Disc Disease

Intervertebral degenerative disk disease (IVDD) is an age-related medical condition where one or more intervertebral discs of the spine undergo degeneration, causing severe neck and back pain. It is a multifactorial disease, but primarily its cause is the loss of soluble proteins from the nucleus pulposus (NP) of the intervertebral discs. NP consists of collagen fibrils and plays a significant role in tissue’s generation and maintenance [45]. One of the major causes of IVDD pathology is the apoptosis of NP cells and the continuous degeneration of the extracellular matrix (ECM). It has been observed that A disintegrin and metalloproteinase with thrombospondin motif 5 (ADAMTS5) and Caspase (Cas3) play significant roles in the deterioration of the ECM and apoptosis, respectively. Dual siRNAs against ADAMTS5 and Cas3 were designed, and in vitro analysis revealed that ATS5-Cas3-siRNA increased the regeneration of ECM in damaged discs and reduced apoptosis of NP cells [46]. The results of this study have suggested that such dual siRNA therapy can be a better way to treat IVDD. Sirtuin 1 (SIRT1) is an NAD-dependent deacetylase that slows down apoptosis in many cells. To understand its role in apoptosis of disc NP cells, siRNA against SIRT1 mRNA was designed and delivered in patients with lumbar disc degenerative disease (LDDD) and lumbar vertebral fracture (LVF) [47]. The results revealed that phosphorylation of AKT serine/threonine kinase 1 (AKT) was significantly reduced in NP cells. The results of this study suggest that SIRT1 plays a significant role in NP cell survival via the AKT anti-apoptotic signaling pathway. Another in vitro study investigated the role of Survivin in LDDD and LVF [48]. Survivin plays a crucial role in the regulation of cell division and in the inhibition of apoptosis in NP cells by blocking Caspase activity. siRNA targeting Survivin was designed and investigated in NP cells of LPDD and LVF patients. The results revealed that Survivin siRNA significantly reduced the proliferation rate of NP cells and increased sensitivity to pro-apoptotic signals. This suggests that Survivin plays a fundamental role in the proliferation and prevention of apoptosis of damaged NP cells, leading to inhibition of disc degenerative episodes. In IVDD, low back pain is the main problem, which is enhanced due to the expression of proinflammatory cytokines such as IL-1β, which are abundantly present in NP cells. SIRT1 supports the regulation of aging and immune responses through anti-apoptotic and anti-catabolic signaling. siRNA against the SIRT1 gene was designed, and its effect was investigated in NP cells, which revealed that SIRT1 siRNA increased IL-1β-induced apoptosis and enhanced the expression of MMPs [49]. NF-kB p65 plays an important role in IVDD pathology. It was observed that mRNA levels of the trigger receptor expressed on myeloid cells-2 (TREM2) are strongly correlated to NF-kBp65 in the NP cells of IVDD patients. TREM2-siRNA was designed and transfected into NP cells, which revealed that it significantly reduced cell apoptosis, enhanced cell proliferation, suppressed proinflammatory cytokines (IL-1β, IL-6, and TNF-α), and downregulated NF-kBp65 expression in NP cells [50]. The results of this study recommend that silencing TREM2 can be an effective strategy for the prevention of IVDD in humans. Mitophagy is a cellular process that plays a critical role in eliminating impaired mitochondria to protect against cellular damage. The Parkin gene (PRKN1) encodes the Parkin protein, which helps in the degradation of non-required proteins by tagging them with a molecule called ubiquitin. To understand Parkin-mediated Mitophagy, a study demonstrated that knock-down of Parkin mRNA by siRNA increased apoptosis and reduced mitochondrial Mitophagy in NP cells of IVDD patients [51]. Lately, for the treatment of IVDD, therapeutic siRNA targeting p65 was mixed with phenylboronic acid-modified G5 polyamidoamine (G5-PBA) and loaded into hydrogels [52]. It was observed that siRNA G5-PBA inhibited the expression of p65/NLRP3 signaling, leading to the attenuation of inflammatory cytokine storms. This study has proposed the efficacy of gene-cell combination therapy for the treatment of IVD regeneration. 

### 3.5. Small Interfering RNA Therapy for Fracture Healing

Fractures due to bone disorders impact substantially skeletal health globally. Worldwide, around 178 million fractures have been observed in 2019, with a future upward trend [1]. Approximately one-tenth of these fractures are either delayed or remain non-union. In the clinical arena of orthopedics, less efficient pharmacological strategies are available for the healing and regeneration of bone fractures. Hence, novel mechanisms are required to accelerate the repair and regeneration of fractures. Some studies have suggested that the plasminogen activation system plays a significant role in bone remodeling and metabolism by removing fibrin and excessive plasmin-associated fibrinolysis [53,54,55]. A siRNA-based study has investigated the role of Plasminogen activator inhibitor-2 (SerpinB2) in fracture healing and repair [56]. SerpinB2 is a serine protease that inactivates tissue plasminogen activator in human bone marrow mesenchymal stem cells (hBMSCs). siRNA was designed to target the SerpinB2 gene and delivered to hBMSCs, which revealed that silencing SerpinB2 significantly increased osteoblast differentiation of hBMSCs and mineralization in vitro and increased β-catenin levels. It was observed and validated that injection of SerpinB2 siRNA locally into the tibial fracture improved fracture healing. The results suggest that SerpinB2 siRNA can effectively promote fracture healing in vivo, making it a potent target for clinical management of fractures. Similarly, to understand the role of Epidermal growth factor-like domain protein-7 (EGFL7) in the bone fracture microenvironment, siRNA targeting EGFL7 was designed and delivered to hBMSCs, and simultaneously, recombinant human (rh) BMSCs were used to evaluate changes in osteogenic differentiation and proliferation of hBMSCs [57]. To determine whether Notch signaling rescues the downregulation of osteogenic differentiation by EGFL7 siRNA in hBMSCs, a γ-secretase inhibitor was used, which blocked Notch1 signaling. The results were confirmed in recombinant mouse EGFL7 for bone repair and regeneration in vivo. It was observed that EGFL7 silencing degrades hBMSC osteogenic differentiation and stimulates Notch/NICD/Hes1 signaling. Interestingly, the EGFL7 knock-down effect of osteoblast inhibition was rescued by Notch1 signaling inhibition. The results of this study suggested that the use of recombinant EGFL7 is a better strategy for fracture healing.

Another study utilized low-intensity pulsed ultrasound (LIPUS) to investigate its fracture healing capabilities mediated by the Hippo signaling pathway [58]. Key transcriptional co-activators of the Hippo pathway are Yes-associated protein1 (YAP) and Transcriptional co-activator with PDZ-binding domain (TAZ), which were knocked down by siRNA and shRNA, respectively, to investigate whether LIPUS can activate YAP/TAZ expression. The results revealed that both of these angiogenic and vascular remodelers were significantly increased after LIPUS treatment. The results of this study have proposed that LIPUS treatment mediates the Hippo pathway to angiogenesis-mediated fracture healing. The Chordin gene (CHRD) is a pleiotropic gene that encodes the Chordin protein that regulates dorsal-ventral tissue differentiation in early embryonic development. Chordin was observed to be highly expressive in fractures. To investigate its role in normal and non-union fracture healing, Chordin siRNA was designed and delivered to hBMSCs using Polyspermine imidazole-4,5-imine (PSI) [59]. The results advocate that Chordin knock-down accelerates hBMSC osteogenesis and bone healing in both in vitro and in vivo, recommending Chordin as a potential therapeutic target for general as well as non-union fracture healing. Survivin plays a significant role in inhibiting apoptosis. To examine its role in degenerative NP cells, especially in lumbar fracture healing (LFH), siRNA was designed to target Survivin and delivered to NP cells in vitro [48]. Survivin siRNA significantly reduced proliferation and migration of NP cells, suggesting it to be an effective strategy for improving fracture healing. Some studies have recommended that activation of P2X purinoceptor 7 (P2X7) receptors by providing extracellular adenosine triphosphates (ATPs) can stimulate proliferation and mineralization of osteoblasts [60,61]. It is proven by a siRNA-based study that shockwave therapy releases ATPs from hMSCs and activates P2X7 receptors, causing hMSCs to stimulate osteoblast differentiation and mineralization, leading to accelerated bone fracture healing [62].

## 4. Mechanisms for siRNA Delivery in Bone Disorders

As an emerging therapeutic intervention for various disorders, siRNA technology has shown tremendous development in recent years. siRNA is a highly specific gene-silencing biomolecule used in genomic medicine, whose invivo and invitro delivery can be performed for the blockage of negative regulators of signaling mechanisms involved in the pathogenesis of various bone diseases. siRNA demonstrates physiochemical characteristics such as large molecular mass and size, negative charges, and instability, which cause hindrance in their protective penetration into the biological membranes to reach target cells both invitro and invivo. The key challenges in delivering siRNA in vivo include premature nuclease breakdown, reticuloendothelial system (RES) clearance, insufficient accumulation at organs or cells of interest, limited tissue penetration, cellular internalization, endosomal escape, and off-target activity [63]. Due to limited drug penetration in bone and inadequate vascular perfusion of bone tissue, siRNA delivery to the bone is even more difficult. Therefore, some putative strategies developed to deliver siRNA for its curative effects on the damaged bone have been discussed. The details of the delivery methods of siRNAs, their respective targets, main findings, species/model, and tissue type used have been given in Table 1.

### 4.1. Cationic Polymer-Based Delivery Systems

Organic polymers, which are cationic in nature, demonstrate characteristics such as lowimmunogenicity and functional groups for essential interactions with cellular components. When cationic polymers are conjugated with biomolecules such as siRNA, these act as nanocarriers for their effective and efficient delivery to the target tissue for their salubrious effects. The earliest cationic polymer-mediated siRNA delivery was reported by Bologna et al. in 2003, in which polyethyleneimine (PEI) was complexed with P2X3 siRNA for its delivery to the hamster ovary cells for the successful knockdown of the P2X3 gene in the ovary cells [64]. The positive charge on these polymers stabilizes the highly negative charge on siRNA, which increases its uptake and prolongs its persistence in the target tissue, evades its lysis by nucleases, and helps in their endolysosomal escape [65]. Cationic polymer-based delivery systems include cationic polymer-derived nanoparticles, spermine-based delivery, Chitosan-mediated delivery, Atelocollagen-mediated delivery, and Albumin-based nanoparticles.

#### 4.1.1. Cationic Polymer-Derived Nanoparticles

In this technique, synthetic biopolymers such as Polylactic-co-glycolic acid (PLGA) are combined with cationic polymers such as PEI to form cationic polymer-derived nanoparticles. PLGA has various attributes, such as biocompatibility, biodegradability, low immunogenicity, and high stability in body fluids. PEI, being cationic, can form non-covalent linkages with highly negative biomolecules such as siRNA, which can lead to their efficient delivery to the target tissue by protecting them from lysis by nucleases, facilitating their tissue internalization, and facilitating their endolysosomal escape to the cytosol [66]. Cytotoxicity and genotoxic effects are noticed when a high concentration of PEI is used due to its high charge density, which can be reduced by its acetylation (Ac), making it safe as a vector for siRNA delivery. An acetylated PLGA-PEI (Ac-PLGA-PEI) nanocomplexgel was designed to deliver Matrix metalloproteinase 2 (MMP-2) siRNA to the PC3 cell line of a human prostate tumor for gene silencing and to the collagen matrix embedded in the human chondrocyte cell line C20A4 for chondrocyte dedifferentiation (a hallmark process of osteoarthritis) in vitro to develop a siRNA therapy for osteoarthritis. PC3 cells and C20A4 cells exhibited efficient cellular uptake of MMP-2 siRNA, effective endosome escape, and successful knockdown of MMP-2 expression, coupled with inhibition of chondrocytic dedifferentiation-related genes, collagen type II alpha 1 chain (COL2A1) and Aggrecan (ACAN). It also prevented matrix breakdown in C20A4 cells when transfected with the MMP-2 siRNA/nanogel complex [66]. RANK siRNA-conjugated PLGA nanoparticles can be embedded into bone augmentation biomaterials such as calcium phosphate cements (CPC), which improve the anchoring of implants around weak osteoporotic bones and allow for early mobilization, which accelerates fracture healing (22951320) [67]. However, these bone cements have been shown to cause symptoms like hypoxemia, hypotension, and sudden loss of consciousness, collectively known as bone cement implantation syndrome (BCIS), in severe trauma cases, specifically hip fractures (37763149) [68].

#### 4.1.2. Spermine-Based Delivery System

Spermine is an endogenous polyamine that is commonly found in human sperm. In the physiological condition, spermine contains a positive charge, which helps stabilize deoxyribonucleic acid (DNA), leading to its condensation in the sperm. Spermine is a cationic polymer that demonstrates characteristics such as biocompatibility, negligible toxicity, and biodegradability. Due to these properties, Spermine is able to conjugate with different groups of polymers to form Polyspermine-based nanocarriers to transport various types of biomolecules, such as siRNA and drugs, to the target tissue [69]. A polyspermine imidazole-4,5-imine (PSI) polyplex was developed by conjugating Chordin siRNA with PSI to deliver Chordin siRNA to the bone-forming mesenchymal stem cells (MSCs) extracted from patients suffering from bone nonunion fracturesin vitro and to the tibial monocortical defect modelin vivo. Efficient cellular uptake, effective knockdown of Chordin expression with no cytotoxicity, and an increase in osteoblastic differentiation leading to osteogenesis and bone regeneration were reported in both MSCs and tibial defects in an experimental rat model [59].

#### 4.1.3. Chitosan-Mediated Delivery System

Chitosan is a cationic polysaccharide formed by the deacetylation of chitin. Being structurally similar to chitin, a primary component of the arthropod exoskeleton, chitosan is known for its low cytotoxicity, biocompatibility, and biodegradability. Chitosan has the ability to combine with drugs and biomolecules like siRNA, which facilitates their endosomal escape to the cytoplasm in the cells and avoids their nuclease-mediated lysis, hence helping in their direct delivery to the target tissue. Chitosan has poor solubility at physiological pH, which can be resolved by binding it to molecules that increase its bio-solubility [70]. A folic acid-coupled novel polysaccharide derivative formed by azidized chitosan conjugated with poly (L-lysine) dendrons (PLLD) was created for the allocation of astrocyte elevated gene-1 (AEG-1) siRNA to the 143B and U20S cell lines of human osteosarcoma in vitro. Efficient transfection of the AEG-1 siRNA/polysaccharide derivative complex into the cell lines, increased cellular uptake, and remarkable knockdown of AEG-1 expression were noted in the 143B and U20S cells. Similar findings, along with anti-metastatic conditions, were reported in the human 143-B cell-infused nude mouse model in vivo [71].

#### 4.1.4. Atelocollagen-Mediated Delivery System

Atelocollagen is a highly purified pepsin-treated Type-I collagen that is derived from the calf dermis. Atelocollagen has low immunogenicity because of its telopeptide-free structure. Due to its similarity with the most commonly found protein in the body, i.e., collagen, Atelocollagen exhibits low cytotoxicity, extreme biocompatibility, and is easily biodegradable [72]. When combined with biomolecules such as siRNA, Atelocollagen supports its effective cellular uptake, resistance to nucleases, and prolonged release of siRNA to the target tissue. A complex consisting of Atelocollagen with EZH2 and p110-α siRNA was formulated to deliver the dual siRNAs to the PC-3M-luc-C6 cell line of human prostate cancerin vitro. The PC-3M-luc-C6 cells reported efficient cellular uptake of both siRNAs and effective suppression of EZH2 and p100-α genes. Similar findings were reported, along with increased persistence in the bone and regression in the metastasis of tumors in the bone tissues without activating non-specific genes that can induce auto-immunity in the body. Absence of interferon-γ and IL-12 expression was detected in the human PC-3M-luc-C6 cell line inoculated in athymic nude mice in vivo [73].

#### 4.1.5. Albumin-Based Nanoparticles

Human serum albumin (HSA) is a water-soluble globular protein that is abundantly present in human plasma. HSA is rich in cysteine residues that are acidic, hence cationic in nature, and form sulfhydryl linkages to bind its three peptide chains for maintaining its tertiary conformation. Moreover, the surface of HSA is rich in these cysteine residues, which participate prominently in the conjugation of a variety of biomolecules, such as siRNA, to HSA molecules. Due to these structural properties, HSA functions as an extraordinary carrier protein in physiological conditions to supply nutrients to the body tissues [74]. When combined with a variety of organic and inorganic molecules, HSA can be engineered into albumin-based nanoparticles, which act as delivery vehicles for siRNA to the target bone tissue. An ultrasound-directed nanodroplet composed of albumin and a perfluorocarbon gas core was designed to encapsulate CTSK siRNA.

It was embedded with alendronate (AL) for their delivery to the human marrow-derived mesenchymal stem cells (hMSCs) for biocompatibility and to the osteoclast precursors for the development of siRNA therapy for osteoporosis. Cytotoxicity was observed to be negligible in hMSCs, and suppression of osteoclastogenesis was reported in the osteoclast precursors in vitro [28].

**Table 1:** siRNA delivery methods for bone repair and regeneration.

S.No.	Delivery Method	Target	MAIN OUTCOME	Species/Model/Tissue Type	Reference
1.	Cationic Polymer-derived nanoparticles (Ac-PLGA-PEI)	MMP-2	Effective silencing of MMP-2 expression, which prevented chondrocyte dedifferentiation.	Human/in vitro/PC3 and C20A4 cell lines of prostate cancer and chondrocytes, respectively.	[66]
2.	Spermine-based polyplexes (PSI)	Chordin	Knockdown of the Chordin gene, which induced osteoblast differentiation and bone regeneration.	Human/in vitro/bone-forming MSCs of nonunion fracture patients. Mouse/in vivo/tibial monocortical defect model.	[59]
3.	Chitosan-mediated delivery method (FA-coupled PLLD and azidized chitosan)	AEG-1	Efficient inhibition of AEG-1 expression provoked anti-invasive effects.	Human/in vitro/143B, and U20S cell lines of osteosarcoma. Mouse/in vivo/143B cell-inoculated nude athymic model.	[71]
4.	Atelocollagen-based delivery system	EZH-2 and p100-α	Knockdown of EZH2 and P100-α genes causes suppression of bone tumors without eliciting an auto-immune response.	Human/in vitro/PC-3M-luc-C6 prostate cancer cell line. Mouse/in vivo/PC-3M-luc-C6 in a nude athymic model.	[73]
5.	Albumin-derived nanodroplets (ultrasound-driven)	CTSK	Repression of CTSK expression, which prompted the inhibition of osteoclastogenesis.	Human/in vitro/MSCs and osteoclast precursors.	[28]
6.	Nanomicelles (LA combined with the cross-linked peptide LACL)	SBREP1	Efficient silencing of the SBREP1 gene evoked anti-metastatic conditions.	Human/in vitro/PC-3 and C4-2B cell lines of BmCRPC. Mouse/in vivo/BALB/c model.	[75]
7.	Liposomes (Lipofectamine RNAiMAX)	mTORC1, C2, RICTOR and RAPTOR	Suppression of respective genes, which promoted ECM formation and halted apoptosis and senescence.	Human/in vitro/disc NP cells derived from IVDD patients who have undergone lumbar interbody fusion surgery.	[76]
8.	Solid lipid nanoparticles (PEGylated)	TNF-α	Decrease in inflammation and joint healing as a result of knockdown of TNF-α gene expression.	Mouse/in vitro and in vivo/J774A.1 macrophage cell line, LPS-induced inflammation, and collagen-induced arthritis model.	[77]
9.	Exosomes (conjugated with bone targeting peptide)	Shn3	Shn3 gene silencing led to upregulation of osteogenesis and increased vascularization.	Mouse/in vitro and in vivo/Raw264.7 and MC3T3-E1 cell lines of macrophages and osteoblast precursors, respectively.	[78]
10.	Titanium Implants (coated with nanoparticle film)	Ckip-1	Effective inhibition of Ckip-1 expression augmented ECM mineralization and osteoblast differentiation.	Human/in vitro/H1299 and MG63 cell lines of lung carcinoma and osteosarcoma, respectively.	[79]
11.	Iron Oxide nanocages (magnetic field directed)	mGluR5	Decline in mGluR5 gene expression reduced anti-proliferative effects.	Human/in vitro/LM7 cell line of osteosarcoma. Mouse/in vitro/OS482 cell line of osteosarcoma.	[80]
12.	Cerium Oxide nanobubbles	CTSK	Silencing of CTSK expression elevated osteogenesis and suppressed osteoclastogenesis.	Human/in vitro/MSCs and osteoclast precursors.	[30]
13.	MBG nanospheres	RANK	Blocking of NF-kB signaling causes repression of osteoclastogenesis.	Mouse/in vitro/Raw264.7 macrophage cell line and osteoclast precursors.	[24]
14.	DSS-6 oligopeptide nanocarriers (complexed with cationic liposomes)	CrkII	Increased bone mass results from effective knockdown of the CrkII gene.	Mouse/in vivo/RANKL-induced bone loss model.	[29]
15.	(Asp) 8 oligopeptide nanoparticles (complexed with HPMA)	sema4D	Reduced expression of sema4D stimulates bone surface remodeling and recovery from bone loss.	Mouse/in vitro/BMSCs and BMMs from the Kunming mouse model. Mouse/in vivo/OVX osteoporotic mouse model.	[81]
16.	Aptamer based nanoparticles	BMP-2	Downregulation of the TNF-α-dependent NF-kB pathway led to the healing of joints and a decrease in edema.	Human/in vitro/MSCs. Mouse/in vivo/curdlan-treated Zap70mut ankylosing spondylitis model.	[82]
17.	Nucleofection (kit with reagents)	PI3Kδ	Repression of PDGF-dependent Rac-1 activation enhanced anti-arthritic effects.	Human/in vitro/FLS cells from OA and RA patients who underwent synovectomy.	[83]
18.	OC/GCA-cross-linked hydrogel	p65	Protection from disc degeneration as a consequence of suppression of NF-kB-mediated NLRP3 inflammasome activation.	Human/in vitro/disc NP cells. Rat/in vivo/NP cells of the IVDD model.	[52]
19.	CdTe/ZnS/3-MPA QDs (combined with β-CD and CKKRGD)	PPARγ	Decrease in PPARγ expression led to an enhancement of osteogenesis.	Human/in vitro/MSCs. Mice/in vivo/hMSCs implanted a nude athymic model.	[84]
20.	Carbon Dots (bioconjugated with sulfo-SMCC)	TNF-α	Suppression of TNF-α-directed pathways, elevated chondrogenesis, and cartilage regeneration in the patellar joint	Rat/in vitro/MSCs from the femur and tibia. Rat/in vivo/MSCs implanted in SD patellar joint defect model.	[85]
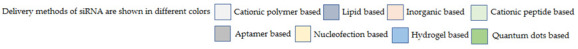					

**Ac-PLGA-PEI:** Acetylated-Poly lactic-co-glycolic acid, polyethyleneimine, **MMP-2:** Matrix metalloproteinase-2, **PSI:** Polyspermine imidazole-4,5-imine, **MSCs:** Mesenchymal stem cells, **FA:** Folic acid, **PLLD:** Poly (L-lysine) dendrons, **AEG:** Astrocyte elevated gene-1, **EZH2:** Enhancer of Zeste homolog 2, **p100-α:** nuclear factor-kappa beta subunit 2, **BmCRPC:** Bone metastatic castration-resistant prostate cancer, **BALB/c:** Bagg albino, **mTORC1:** Mammalian target of rapamycin complexes 1, **mTORC2:** Mammalian target of rapamycin complexes 2, **RICTOR:** rapamycin-insensitive companion of mTOR, **RAPTOR:** regulatory-associated protein of mTOR complexes 1 and 2 **ECM:** Extracellular matrix, **NP:** Nucleus pulposus cells, **IVDD:** Intervertebral disc degeneration, **PEG:** Polyethylene glycol, **TNF-α:** Tumor necrosis factor-alpha, **LPS:** Lipopolysaccharide, **Shn3:** Schnurri-3, **Ckip-1:** Casein kinase 2 interacting protein-1, **mGluR5:** metabotropic glutamate receptor type 5, **MBG:** Mesoporous bioactive silica galss, **RANK:** Receptor activator of nuclear factor kappa beta, **NF-kB:** Nuclear factor-kappa beta, **DSS-6:** (Aspartate-Serine-Serine) 6 tripeptide, **CrkII:** C10 regulator of kinase II; **RANKL:** Receptor activator of nuclear factor kappa beta ligand; **Asp:** Aspartate, **HPMA:** N-(2-hydroxypropyl)methacrylamide, **sema4D:** Semaphorin 4D, **BMSCs:** bone marrow stromal cells, **BMMs:** bone-marrow-derived monocyte/macrophage precursor cells, **OVX:** Ovariectomized mice, **BMP-2:** Bone morphogenetic protein-2; **PI3Kδ:** Phosphoinositide 3-kinase delta; **PDGF:** Platelet-derived growth factor, **Rac-1:** Ras-related C3 botulinum toxin substrate 1 **FLS:** Fibroblast-like synoviocytes, **OA:** Osteoarthritis, **RA:** Rheumatoid arthritis, **OG/GCA:** Girard reagent T-modified oxidized dextran/adipic acid dihydrazide (ADH)-grafted catechol-coupled gelatin, **p65:** Nuclear factor-kappa beta p65 subunit, **NLRP3:** nucleotide-binding domain, leucine-rich–containing family, pyrin domain–containing-3, **CdTe:** Cadmium telluride, **ZnS:** Zinc sulphide, **3-MPA:** 3-Mercaptopropionic acid, **QDs:** Quantum dots, **β-CD:** Beta-cyclodextrin, **CKKRGD:** Cysteine-Lysine-Arginine-Glycine-Aspartate Peptide, **PPARγ:** Peroxisome proliferator-activated receptor gamma; **SMCC:** Sulfosuccinimidyl-4-(N-maleimidomethyl) cyclohexane-1-carboxylate; **SD:** Sprague-Dawley rat model.

### 4.2. Lipid-Based Delivery Systems

Lipids, one of the most multifaceted molecules, are commonly used as drug carriers for curing bone diseases. The amphiphilic behavior of lipids contributes to the formation of micelles and layers, which can encapsulate biomolecules like siRNA (lipoplexes), hence can be used as vehicles for their direct delivery to the target tissue [86]. The earliest use of lipids as a delivery vehicle for siRNA to the target tissue was reported by Sorenson et al. (2003), in which siRNA was delivered to silence TNF-α using 1,2-dioleoyl-3-trimethylammonium-propane (DOTAP) cationic liposomes to murine peritoneal macrophages in vitro and to the Bagg Albino (BALB/c) mouse model in vivo, which resulted in the suppression of TNF-α gene expression in both murine macrophages in vitro and in vivo [87]. Lipid-delivery systems include nanomicelles and liposomes.

#### 4.2.1. Nanomicelles-Based Delivery System

Nanomicelles are colloidal structures composed of amphiphilic monomers with a small hydrophobic head, which lines the interior of the micelle, and a long hydrophilic tail, which makes the exterior of the micelle in an aqueous medium [88]. When complexed with different types of polymers, nanomicelles act as carriers for transporting siRNA to the target bone tissue. A bio-mimetic system was formulated by combining lipoic acid (LA) with cross-linked peptide-lipoic acid micelle (LACL) to deliver sterol regulatory element-binding protein-1 (SREBP1) siRNA and docetaxel (DTX) to the cell lines PC-3 and C4-2B of human bone metastatic castration-resistant prostate cancer (BmCRPC) in vitro. Effective silencing of SREBP-1 and stearoyl Co-A desaturase 1 (SCD-1; down regulatory gene of SBREP1), along with increased uptake of siRNA by the tumor cells, combinatorial attack on bone cancer, and anti-proliferating and anti-invasive effects of both DTX and siRNA, were observed in the two cell typesin vitro. Analogous observations accompanied by the restoration of DTX sensitivity to cancer by inhibiting dysregulated lipid metabolism, a key feature of many metastatic cancers, were seen in the BALB/c mice model in vivo [75]. 

#### 4.2.2. Liposomes-Based Delivery Systems

Liposomes are globular lipid vesicles formed from one or more lipid layers as a result of the emulsification of lipids in an aqueous medium [89]. Liposomes are a multifaceted delivery vehicle for a variety of drugs and biomolecules, such as siRNA, because of their similar composition to the membranes of cells and organelles, which aid in direct delivery to the target site. Doxil was the first FDA-approved nano-drug based on a cationic liposome-mediated delivery system for treating a variety of cancers [90]. Liposomes are composed of different types of lipids (both natural and synthetic) and can be complexed with different types of cationic polymers and cationic peptides. This makes them an excellent nano-delivery system to transport siRNA into the targeted bone tissue, as they can prompt their efficient penetration into the cells and nuclear localization without causing auto-immunity. It entraps siRNA within them by encapsulation to avoid interaction with serum proteins. A cationic liposome-transfecting agent, Lipofectamine^TM^ 3000, was fused with high-temperature serine protease A (HtrA2) siRNA into the fibroblast-like synoviocytes (FLSs) derived from individuals suffering from rheumatoid arthritis and osteoarthritis with articular replacement surgery in vitro. Suppression of the HtrA2 gene along with inhibition of expression of various pro-inflammatory biomarkers like IL-1β, TNF-α, CCL-2, IL-6, and IL-8 was observed without exhibiting any cytotoxicity in the FLS cells in vitro [91]. A lipofectamine RNAiMAX reagent was employed to encapsulate siRNAs to silence mammalian targets of rapamycin complexes 1 and 2 (mTORC1 and mTORC2), regulatory-associated proteins of mTOR complexes 1 and 2 (RAPTOR), and rapamycin-insensitive companions of mTOR (RICTOR) of the mTOR signaling pathway. These were delivered to the human disc NP cell line obtained from the SV40 recombinant adenovector and to the human disc nucleus NP cells obtained from patients suffering from intervertebral disc degeneration (IVDD) who had undergone lumbar interbody fusion surgery. The results revealed that it caused effective knockdown of the respective genes, differential activation of the Akt gene, stimulation of autophagy by IL-1β, protection against apoptosis and senescence, a decrease in the expression of matrix catabolizing proteins, and the maintenance of theextracellular matrix (ECM) in both types of human disc NP cells [76].

### 4.3. Aptamer-Based Delivery System

Aptamers are a type of nucleic acid sequence that is generally composed of a single-stranded RNA or DNA that is 25–30 nt bases long. Aptamers are arranged in a distinct tertiary conformation and conjugated to a variety of nanoparticles to design an efficient non-viral vector system for drug delivery [63]. These aptamer-modified nanoparticles can recognize and bind to their target molecule, such as siRNA, with high specificity and affinity, thereby facilitating their nuclear localization and enhanced cellular uptake by the target cells. An anti-ROS osteoblast-specific, CH-6 aptamer-modified manganese ferrite nanoparticle system was constructed to deliver bone morphogenetic protein-2 (BMP2) siRNA to human mesenchymal stem cells (hMSCs)in vitro. An increase in cellular uptake, enhanced cell viability along with negligible cytotoxic effects, successful endolysosomal escape, decrease in reactive oxygen species (ROS) levels, inhibition of the TNF-α-mediated NF-kB signaling pathway, and repression in the expression of osteoblastic genes (BMP2, Runt-related transcription factor 2 (Runx2), and osteopontin (OPN))were evident [82]. Similar inferences, along with a reduction in redness and edema in the ankle and healing of joints, were reported in the in vivo curdlan-treated Zap70^mut^ mouse model of ankylosing spondylitis (AS) [82]. 

### 4.4. Inorganic-Based Delivery Systems

Several classes of inorganic elements and inorganic compounds are combined with various types of nanoparticles to develop non-viral vectors for effective drug delivery [92]. These inorganicnanoparticles can be complexed with a variety of therapeutic biomolecules, like siRNA, for delivery to damaged bone tissues. Inorganic-based delivery systems include titanium implants, iron-oxide nanocages, cerium-oxide nanoparticles, and Silica-based nanoparticles.

#### 4.4.1. Titanium Implant-Based Delivery System

Titanium oxide is an excellent implant material because of its biocompatibility, corrosive resistance, and low elasticity. The titanium surface forms attachment to the bones via a process called osseointegration, in which biomolecules are absorbed on the titanium oxide layer along with the migration of osteoblasts and other immune cells into the implant [93]. This process of osseointegration makes titanium implants an outstanding vehicle to transport biomolecules and drugs to their target sites. When these titanium implants are embedded with nanopolymers or nanoparticles encapsulating siRNA, they can be used as carriers for siRNA in the target bone tissue. A titanium implant coated with a multi-layered film made of sodium hyaluronate and chitosan nanoparticles encapsulating siRNA by a layer-by-layer (LbL) process was constructed to deliver green fluorescent protein (GFP) siRNA and casein kinase-2 interacting protein-1 (Ckip-1) siRNA to the H1299 cell line of human lung carcinoma and the MG63 human cell line of osteosarcoma, respectively, in vitro. Decreased expression of the GFP gene with negligible cytotoxicity was noticed in H1299 cells, whereas successful knockdown of the Ckip-1 gene, augmented alkaline phosphatase (ALP) activity, enhanced collagen secretion, an increase in extracellular matrix (ECM) mineralization, and hence the differentiation of osteoblasts were observed in MG63 cells in vivo [79].

#### 4.4.2. Iron Oxide Nanocage-Based System

Iron Oxide (IO) nanoparticles are spherical cage-like structures that are composed of magnetite (Fe_3_O_4_) and maghemite (γ-Fe_3_O_4_) forms of iron oxide, which are supramagnetic in nature. These IO-nanocages are chemically inert and also exhibit properties like strong magnetic and catalytic behavior, biocompatibility with low cytoxicity, and high stability in biological fluids, which makes them exceptional vectors for drug/biomolecule (siRNA) delivery to the required tissues, such as bone [94]. When placed under an alternating magnetic field, IO-nanocages exhibit Brownian motion, which helps in their endosomal escape and direct delivery of siRNA into the cytoplasm of the target cell, facilitating their efficient delivery [95]. A magnetically driven nanocarrier was engineered from IO-nanocages to deliver metabotropic glutamate receptor type 5 (mGluR5) siRNA to the human and mouse metastatic osteosarcoma cell lines LM7 and OS482, respectively, in vitro, subjected to alternating magnetic fields (AMFs). Proper cellular uptake by Clathrin and caveolae-mediated endocytosis in LM7 and OS482 cells, respectively, and silencing of the mGluR5 gene leading to inhibition of tumor growth and metastasis were observed in both LM7 and OS482 cells [80].

#### 4.4.3. Cerium Oxide Nanoparticles

Cerium oxide nanoparticles (CeNPs), also known as nanoceria, have a cubical structure consisting of cerium ions existing in +3 and +4 states, which contribute totheir anti-oxidant properties that mimic superoxide dismutase (SOD) and phosphatase enzyme [96]. Moreover, cerium oxide (CeO_2_) exhibits ferromagnetic behavior and is non-toxic to living cells. When complexed with different biomolecules, such as siRNA, via encapsulation within their lattice, CeNPs can be employed as nanocarriers for the optimal delivery of these molecules to the target bone tissue. An ultrasound-mediated nanobubble delivery system was engineered, for which nanobubbles were created from cerium oxide nanoparticles (CeNPs) complexed with albumin and perfluorohexane. These were loaded with CTSK siRNA for its delivery to the human bone-marrow-derived mesenchymal stem cells (hMSCs) for gene silencing and to the human osteoclast precursors for bone regeneration invitro. Efficient cellular uptake and successful knockdown of CTSK gene expression with insignificant cytotoxicity were observed in both hMSCs and osteoclast precursors, accompanied by an increase in the rate of osteogenic differentiation, enhanced mineralization of the extracellular matrix, and a down-regulation of osteoclastogenesis in the osteoclast precursor cells [30].

#### 4.4.4. Silica-Based Nanoparticles

Porous silicon is procured by an electrochemical etching of monocrystalline silicon in hydrofluoric acid. Silica, also known as silicon dioxide, is obtained from porous silicon, which is known for its physiochemical properties such as tunable pore size, porosity, biocompatibility, bioinert behavior, and biodegradability [97]. Porous silica nanoparticles facilitate the bioavailability and persistence of the drug for a longer period of time, reduce the toxicity of the drug, and provide precise drug targeting [97]. Because of these characteristics, when combined with a variety of drugs and biomolecules, such as siRNA, porous silica nanoparticles can be exploited as a nanocarrier system for these molecules in the tissue of interest.A mesoporous bioactive glass (MBG) nanosphere composed of silica nanoparticles was constructed to deliver receptor activator of nuclear factor-kappa beta (RANK) siRNA to the monocyte macrophage cell line RAW 264.7 of mice and osteoclast precursors in vitro to design a therapy for osteoporosis. Efficient intracellular uptake without any cytotoxicity, successful suppression of RANK gene expression, and down-regulation of osteoclastic genes: proto-oncogene AP-1 transcription factor subunit 1 (c-fos), Cathepsin K, triiodothyronine receptor auxiliary protein (TRAP), and nuclear factor of activated T-cells 1 (NFATc1) were observed, leading to the inhibition of osteoclastogenesis in both types of cells [24].

### 4.5. Nucleofection-Based Delivery System

Nucleofection is an electroporation-mediated transfection technique that was invented by the biotechnology company Amaxa. Nucleofection involves the use of a specific voltage generator by a device called a nucleofactor along with cell-specific nucleofection reagents to deliver therapeutic biomolecules such as siRNA directly into the cells of the target tissue. Nucleofection is beneficial over lipid-based transfection due to its non-toxicity towards cell types and non-activation of interferons, and it is best for transfection of difficult-to-transfect cell lines such as primary and non-adherent cell lines. A nucleofection kit (developed by Amaxa Co.) was employed to deliver phosphoinositide 3-kinase delta (PI3Kδ) siRNA to the fibroblast-like synoviocytes (FLS) derived from the synovial tissue of osteoarthritis (OA) and rheumatoid arthritis (RA) patients who had undergone synovectomy. Effective knockdown of the PI3Kδ gene results in the inhibition of platelet-derived growth factor (PDGF)-dependent activation of Ras-related C3 botulinum toxin substrate 1 (Rac1), halting the reorganization of the actin cytoskeleton. It ultimately leads to the suppression of the migration and invasion of OA/RA-FLS, which imparts anti-inflammatory and anti-arthritic effects on OA/RA-FLS treated with PI3Kδ siRNA [83].

### 4.6. Quantum Dot-Based Delivery System

Quantum dots (QDs) are inorganic semiconductors that are synthesized by using metal ions and colloid stabilizers, whose surfaces can be modulated through shape control, surface coating, and surface functionalization. These properties help in their local targeting and efficient cellular uptake, but QDs exhibit cytotoxicity in higher quantities. When QDs are combined with other polymers, it lowers their toxicity and makes them biocompatible and biodegradable, ultimately establishing QD nanoparticles as efficient nanocarriers for the delivery of drugs and biomolecules such as siRNA to the target bone tissue, which can help in developing therapies for bone defects [98,99]. A multifunctional QD nanoparticle delivery system was engineered by first creating cadmium telluride (CdTe)/Zinc sulphide (ZnS)/3-mercaptopropionic acid QDs. These are conjugated with β-cyclodetrin (β-CD) and Cys-Lys-Lys-Arg-Gly-Asp (CKKRGD) peptide to deliver dexamethasone (Dex) and fluorescein amidite (FAM)-labelled peroxisome proliferator-activated receptor gamma (PPARγ) siRNA to the human mesenchymal stem cells (hMSCs) in vitro and hMSCs implanted in an athymic nude mouse model in vivo. Efficient knockdown of PPARγ and RUNX 2 genesleading to osteoblast proliferation and differentiation without any toxicity was reported in vitro, and similar results were confirmed by the in vivo model [84].

### 4.7. Hydrogel-Based Delivery System

Hydrogels are constructed by using various types of cationic and lipid-based polymers that have a high water-absorbing capacity. Hydrogels exhibit characteristics such as biocompatibility, lowtoxicity to the cells, and biodegradability, depending on the polymers they are composed of. These hydrogels can be used as nanocarriers for delivering drugs and biomolecules like siRNA to the target tissue. The earliest use of hydrogel for the delivery of siRNA was reported by San Juan et al. in 2009, in which cationized-pullulan-basedhydrogel was employed to deliver MMP-2 siRNA to the balloon-injured carotid arteries of the rabbit model for atherosclerosis. This led to the successful knockdown of MMP-2 gene expression [100]. Hydrogels facilitate the direct delivery of the siRNA to the target tissue and protect siRNA from innateimmunity [101]. AGirard reagent T-modified oxidized dextran/adipic acid dihydrazide (ADH)-grafted catechol-coupled gelatin(OG/GCA)-cross-linked hydrogel was constructed and loaded with Phenylboronic acid-modified G5 (PAMAM-G5)/p65siRNA complex to deliver p65 siRNA to the human NP cells in vitro for developing treatment for inter-vertebral disc degeneration (IVDD). It was observed that this method resulted in efficient knockdown of p65, leading to the down-regulation of NF-kB signaling and the NLRP3 inflammasome without any cytotoxicity, along with mineralization of the ECM and a delay in IVD degeneration in NP cells [52]. Similar results were confirmed by performing the same experiments on a rat model of IVDD in vivo [52].

## 5. A Combinatorial Approach to siRNA Therapy for Various Bone Disorders

A newer strategy, i.e., a combination of chemical drugs and siRNA loaded onto various types of nanoformulations, has been developed as an efficient therapy to treat bone diseases. This dual therapy approach comes with a lot of benefits due to the synergistic effect between drugs and siRNA on the target tissue. The nanoparticle formulations are developed in such a way that they minimize the cytotoxic effects of drugs, off-target silencing via selective accumulation, and suppression of auto-immune reactions in the body due to their non-recognition by TLRs [92]. Similarly, another study reported a dual approach of PEI-modified PLGA nanoparticles to deliver dexamethasone and anti-COX-2 siRNA to the human chondrocyte cell line C28/12, in which rheumatoid arthritis (RA) conditions were induced by TNF-α treatment. Effective silencing of COX-2 expression without any cytotoxicity attenuated expression of pro-inflammatory markers, microsomal prostaglandin E synthase-1(mPGES-1) and inducible nitric oxide synthase (iNOS), along with reduced expression of apoptotic markers, capase-3 and annexin-V [102]. Multiple in vitro studies for developing an array of delivery systems for siRNA and dual drug/siRNA to the bone have been conducted using various cell lines derived from humans to develop therapy for various bone defects, whereas in vivo siRNA studies are still limited.

## 6. Clinical Implications and Future Strategies

The therapeutic utility of RNAi-mediated siRNA has been harnessed for the treatment of several diseases owing to its straightforward mechanism. On the basis of target sequence, siRNA recognizes them and regulates gene function by targeting the gene responsible for their expression. Nonetheless, efficient delivery of siRNA to the target organ and its successful uptake are still to be improved to yield the expected results. Despite the FDA approval of five siRNA drugs for the treatment of hereditary transthyretin-induced amyloidosis with polyneuropathy, hypercholesterolemia, primary hyperoxaluria type 1, and hepatic porphyria, their usefulness for other diseases and the development of new siRNA-based therapeutics are intricate and challenging [103]. 

Several approaches are in use to deliver siRNA, combinations of siRNA with drugs, and dual siRNAs to the target, but many problems exist that must be addressed to make these more effective. First of all, siRNA therapy can be ineffective and unsafe as it may trigger immune reactions, which are very complex to identify and exhibit the worst outcomes. siRNAs are generally degraded by RNAses and phosphatases [104]. Moreover, unmodified naked siRNAs are not only degraded but also recognized by Pathogen-associated molecular patterns (PAMPs) and Danger-associated molecular patterns (DAMPs), which initiate an autoimmune response and inflammatory signaling. To rectify these issues, chemical modifications of the phosphodiester backbone, including ribose, phosphate, and base modifications, are carried out for its better interaction with RISC components and to escape their recognition. Replacing adenosine with N^6^-methyl adenosine in the guide strand of siRNA can evade its recognition by PAMPs and DAMPs [105]. Similarly, addition of 2′-O-Me Uridine and Guanosine to the sense strand of siRNA inhibits inflammatory signaling [106]. Lysosomal function inhibitors such as Bafilomycin and Chloroquine may reduce the recognition of siRNA by toll-like receptors (TLRs) without compromising the effect of siRNA [107]. Secondly, siRNAs are negatively charged and are not able to cross membranes. The half-life of naked siRNA is only 10–15 min, and because of their low molecular weight, they are eliminated by kidneys [108]. To overcome this issue, siRNAs are fused with or encapsulated into liposome/lipid nanoparticles so that they may penetrate the phospholipid bilayers of the cell. Most importantly, the siRNAs expected for on-target silencing also induce many types of off-target silencing. This unexpected and unforeseen off-target activity of specific siRNAs can lead to different outcomes and unwanted toxic reactions. To avoid this problem, chemical modification of the 2′-O-methyl of a single position on the siRNA guide strand is utilized to significantly reduce off-target silencing. This seed region modification of the guide strand is combined with a 2′-O-methyl modification of the passenger strand, which reduces its activity, leading to a significant reduction in off-target silencing. Additionally, increasing copies of the siRNAs (pooling) can mitigate the silencing of unintended transcripts because competition among siRNAs in the pool for on-target silencing reduces off-target transcript binding [109].

By rectifying inefficiencies in siRNA design, regulating efficient siRNA delivery, controlling on-target binding sensitivity, and minimizing associated risks, siRNA therapy can be a promising strategy for several formidable diseases, including bone disorders. The utility of prophylactic application of siRNA to target genomic RNA (gRNA) of SARS-CoV-2 to inhibit replication before the initiation of translation has already confirmed its astounding importance and impact on the field of Genomic Medicine [110]. The availability of gene-specific silencer-select siRNAs and stealth-RNAi siRNAs for effective knockdown with high specificity and less cellular toxicity has facilitated the scientists’ development of improved versions of siRNA therapy. All five siRNA drugs approved so far are siRNA conjugates with N-acetylgalactosamine (GalNAc), which target hepatocytes (liver cells). However, the design and delivery of extra-hepatic siRNAs to other organs, treating undruggable diseases, and developing siRNA-based personalized medicine for multiple disorders remain challenges for the researchers working in this field of genomic medicine.

## 7. Conclusions

The increasing prevalence of musculoskeletal abnormalities and bone fractures globally has engrossed the attention of scientists to explore innovative strategies for the management and effective bone regeneration and healing. While traditional approaches such as bone grafts and growth-promoting factors have shown promising results, their limitations, especially in invoking allergic reactions, unintended infection, bleeding, nerve damage, and inflammation, necessitate a paradigm shift in therapeutic interventions. The advent of RNA interference (RNAi) has revolutionized our approach to addressing complex diseases, and its application in bone disorders holds immense potential. RNAi, specifically small interference RNA (siRNA), has emerged as a promising tool for gene silencing, offering a sequence-specific mechanism to target the underlying causes of bone degeneration and deformities. The FDAs approval of siRNA-based drugs has highlighted the clinical relevance and potential of this technology.

The present review has concluded that in the quest for practical solutions to address the intricate challenges of bone disorders and their prevention, the exploration of siRNA-based therapeutics stands as a beacon of hope. The continued synergy between innovative ideas and their executions with advanced technology holds the key to unlocking the full potential of siRNA in reshaping the landscape of musculoskeletal health. As we navigate this frontier, collaboration among researchers, clinicians, and industry stakeholders will be paramount to accelerating the development and deployment of siRNA-based interventions for the benefit of individuals suffering from bone disorders and fractures.

## References

[1] GBD 2019 Fracture Collaborators (2021). Global, Regional, and National Burden of Bone Fractures in 204 Countries and Territories, 1990–2019: A Systematic Analysis from the Global Burden of Disease Study 2019. Lancet Healthy Longev..

[2] GBD 2017 DALYs and HALE Collaborators (2018). Global, Regional, and National Disability-Adjusted Life-Years (DALYs) for 359 Diseases and Injuries and Healthy Life Expectancy (HALE) for 195 Countries and Territories, 1990–2017: A Systematic Analysis for the Global Burden of Disease Study 2017. Lancet.

[3] Singh M., Singh B., Sharma K., Kumar N., Mastana S., Singh P. (2023). A Molecular Troika of Angiogenesis, Coagulopathy and Endothelial Dysfunction in the Pathology of Avascular Necrosis of Femoral Head: A Comprehensive Review. Cells.

[4] Ren Y., Liu B., Feng Y., Shu L., Cao X., Karaplis A., Goltzman D., Miao D. (2011). Endogenous PTH Deficiency Impairs Fracture Healing and Impedes the Fracture-Healing Efficacy of Exogenous PTH(1-34). PLoS ONE.

[5] Lo Sicco C., Tasso R. (2017). Harnessing Endogenous Cellular Mechanisms for Bone Repair. Front. Bioeng. Biotechnol..

[6] Peng Y., Li J., Lin H., Tian S., Liu S., Pu F., Zhao L., Ma K., Qing X., Shao Z. (2021). Endogenous Repair Theory Enriches Construction Strategies for Orthopaedic Biomaterials: A Narrative Review. Biomater. Transl..

[7] Chen Y.G., Hur S. (2022). Cellular Origins of dsRNA, Their Recognition and Consequences. Nat. Rev. Mol. Cell Biol..

[8] Hammond S.M., Caudy A.A., Hannon G.J. (2001). Post-Transcriptional Gene Silencing by Double-Stranded RNA. Nat. Rev. Genet..

[9] Mittal V. (2004). Improving the Efficiency of RNA Interference in Mammals. Nat. Rev. Genet..

[10] Alshaer W., Zureigat H., Al Karaki A., Al-Kadash A., Gharaibeh L., Hatmal M.M., Aljabali A.A.A., Awidi A. (2021). siRNA: Mechanism of Action, Challenges, and Therapeutic Approaches. Eur. J. Pharmacol..

[11] McCaffrey A.P., Meuse L., Pham T.-T.T., Conklin D.S., Hannon G.J., Kay M.A. (2002). RNA Interference in Adult Mice. Nature.

[12] Song E., Lee S.-K., Wang J., Ince N., Ouyang N., Min J., Chen J., Shankar P., Lieberman J. (2003). RNA Interference Targeting Fas Protects Mice from Fulminant Hepatitis. Nat. Med..

[13] Weng Y., Xiao H., Zhang J., Liang X.-J., Huang Y. (2019). RNAi Therapeutic and Its Innovative Biotechnological Evolution. Biotechnol. Adv..

[14] Sardh E., Harper P., Balwani M., Stein P., Rees D., Bissell D.M., Desnick R., Parker C., Phillips J., Bonkovsky H.L. (2019). Phase 1 Trial of an RNA Interference Therapy for Acute Intermittent Porphyria. N. Engl. J. Med..

[15] Sas D.J., Magen D., Hayes W., Shasha-Lavsky H., Michael M., Schulte I., Sellier-Leclerc A.-L., Lu J., Seddighzadeh A., Habtemariam B. (2022). Phase 3 Trial of Lumasiran for Primary Hyperoxaluria Type 1: A New RNAi Therapeutic in Infants and Young Children. Genet. Med..

[16] Schett G., Loza M.J., Palanichamy A., FitzGerald O., Ritchlin C., Bay-Jensen A.-C., Nielsen S.H., Gao S., Hsia E.C., Kollmeier A.P. (2022). Publisher Correction to: Collagen Turnover Biomarkers Associate with Active Psoriatic Arthritis and Decrease with Guselkumab Treatment in a Phase 3 Clinical Trial (DISCOVER-2). Rheumatol. Ther..

[17] Friedrich M., Aigner A. (2022). Therapeutic siRNA: State-of-the-Art and Future Perspectives. BioDrugs.

[18] Wang S., Wei X., Sun X., Chen C., Zhou J., Zhang G., Wu H., Guo B., Wei L. (2018). A Novel Therapeutic Strategy for Cartilage Diseases Based on Lipid Nanoparticle-RNAi Delivery System. Int. J. Nanomed..

[19] Yi X., Liu J., Cheng M.-S., Zhou Q. (2021). Low-Intensity Pulsed Ultrasound Inhibits IL-6 in Subchondral Bone of Temporomandibular Joint Osteoarthritis by Suppressing the TGF-Β1/Smad3 Pathway. Arch. Oral Biol..

[20] Maurer M.S., Kale P., Fontana M., Berk J.L., Grogan M., Gustafsson F., Hung R.R., Gottlieb R.L., Damy T., González-Duarte A. (2023). Patisiran Treatment in Patients with Transthyretin Cardiac Amyloidosis. N. Engl. J. Med..

[21] Tang S., Tang Q., Jin J., Zheng G., Xu J., Huang W., Li X., Shang P., Liu H. (2018). Polydatin Inhibits the IL-1β-Induced Inflammatory Response in Human Osteoarthritic Chondrocytes by Activating the Nrf2 Signaling Pathway and Ameliorates Murine Osteoarthritis. Food Funct..

[22] Desancé M., Contentin R., Bertoni L., Gomez-Leduc T., Branly T., Jacquet S., Betsch J.-M., Batho A., Legendre F., Audigié F. (2018). Chondrogenic Differentiation of Defined Equine Mesenchymal Stem Cells Derived from Umbilical Cord Blood for Use in Cartilage Repair Therapy. Int. J. Mol. Sci..

[23] Salari N., Ghasemi H., Mohammadi L., Behzadi M.H., Rabieenia E., Shohaimi S., Mohammadi M. (2021). The Global Prevalence of Osteoporosis in the World: A Comprehensive Systematic Review and Meta-Analysis. J. Orthop. Surg. Res..

[24] Kim T.-H., Singh R.K., Kang M.S., Kim J.-H., Kim H.-W. (2016). Inhibition of Osteoclastogenesis through siRNA Delivery with Tunable Mesoporous Bioactive Nanocarriers. Acta Biomater..

[25] Chen H., Xing J., Hu X., Chen L., Lv H., Xu C., Hong D., Wu X. (2017). Inhibition of Heat Shock Protein 90 Rescues Glucocorticoid-Induced Bone Loss through Enhancing Bone Formation. J. Steroid Biochem. Mol. Biol..

[26] Guo B., Zhang B., Zheng L., Tang T., Liu J., Wu H., Yang Z., Peng S., He X., Zhang H. (2014). Therapeutic RNA Interference Targeting CKIP-1 with a Cross-Species Sequence to Stimulate Bone Formation. Bone.

[27] Mora-Raimundo P., Lozano D., Manzano M., Vallet-Regí M. (2019). Nanoparticles to Knockdown Osteoporosis-Related Gene and Promote Osteogenic Marker Expression for Osteoporosis Treatment. ACS Nano.

[28] Shar A., Aboutalebianaraki N., Misiti K., Sip Y.Y.L., Zhai L., Razavi M. (2022). A Novel Ultrasound-Mediated Nanodroplet-Based Gene Delivery System for Osteoporosis Treatment. Nanomedicine.

[29] Seong S., Vijayan V., Kim J.H., Kim K., Kim I., Cherukula K., Park I.-K., Kim N. (2023). Nano-Formulations for Bone-Specific Delivery of siRNA for CrkII Silencing-Induced Regulation of Bone Formation and Resorption to Maximize Therapeutic Potential for Bone-Related Diseases. Biomater. Sci..

[30] Sotoudeh Bagha P., Kolanthai E., Wei F., Neal C.J., Kumar U., Braun G., Coathup M., Seal S., Razavi M. (2023). Ultrasound-Responsive Nanobubbles for Combined siRNA-Cerium Oxide Nanoparticle Delivery to Bone Cells. Pharmaceutics.

[31] Kitajima H., Sakamoto T., Horie T., Kuwano A., Fuku A., Taki Y., Nakamura Y., Tanida I., Sunami H., Hirata H. (2023). Synovial Fluid Derived from Human Knee Osteoarthritis Increases the Viability of Human Adipose-Derived Stem Cells through Upregulation of FOSL1. Cells.

[32] Zhang C., Yu W., Huang C., Ding Q., Liang C., Wang L., Hou Z., Zhang Z. (2019). Chrysin Protects Human Osteoarthritis Chondrocytes by Inhibiting Inflammatory Mediator Expression via HMGB1 Suppression. Mol. Med. Rep..

[33] Yan H., Duan X., Pan H., Akk A., Sandell L.J., Wickline S.A., Rai M.F., Pham C.T.N. (2019). Development of a Peptide-siRNA Nanocomplex Targeting NF- κB for Efficient Cartilage Delivery. Sci. Rep..

[34] Chen X., Liu Y., Wen Y., Yu Q., Liu J., Zhao Y., Liu J., Ye G. (2019). A Photothermal-Triggered Nitric Oxide Nanogenerator Combined with siRNA for Precise Therapy of Osteoarthritis by Suppressing Macrophage Inflammation. Nanoscale.

[35] Luo P., Peng S., Yan Y., Ji P., Xu J. (2020). IL-37 Inhibits M1-like Macrophage Activation to Ameliorate Temporomandibular Joint Inflammation through the NLRP3 Pathway. Rheumatology.

[36] Zhang Z.-J., Hou Y.-K., Chen M.-W., Yu X.-Z., Chen S.-Y., Yue Y.-R., Guo X.-T., Chen J.-X., Zhou Q. (2023). A pH-Responsive Metal-Organic Framework for the Co-Delivery of HIF-2α siRNA and Curcumin for Enhanced Therapy of Osteoarthritis. J. Nanobiotechnol..

[37] Chen D., Zeng S., Huang M., Xu H., Liang L., Yang X. (2017). Role of Protein Arginine Methyltransferase 5 in Inflammation and Migration of Fibroblast-like Synoviocytes in Rheumatoid Arthritis. J. Cell. Mol. Med..

[38] Zou Y., Zeng S., Huang M., Qiu Q., Xiao Y., Shi M., Zhan Z., Liang L., Yang X., Xu H. (2017). Inhibition of 6-Phosphofructo-2-Kinase Suppresses Fibroblast-like Synoviocytes-Mediated Synovial Inflammation and Joint Destruction in Rheumatoid Arthritis. Br. J. Pharmacol..

[39] Wang X., Wang X., Sun J., Fu S. (2018). An Enhanced RRM2 siRNA Delivery to Rheumatoid Arthritis Fibroblast-like Synoviocytes through a Liposome-protamine-DNA-siRNA Complex with Cell Permeable Peptides. Int. J. Mol. Med..

[40] Song B., Li X., Xu Q., Yin S., Wu S., Meng X., Huang C., Li J. (2020). Inhibition of BMP3 Increases the Inflammatory Response of Fibroblast-like Synoviocytes in Rheumatoid Arthritis. Aging.

[41] Zhao M., Zhu T., Chen J., Cui Y., Zhang X., Lee R.J., Sun F., Li Y., Teng L. (2021). PLGA/PCADK Composite Microspheres Containing Hyaluronic Acid-Chitosan siRNA Nanoparticles: A Rational Design for Rheumatoid Arthritis Therapy. Int. J. Pharm..

[42] Zou Y., Shen C., Shen T., Wang J., Zhang X., Zhang Q., Sun R., Dai L., Xu H. (2021). LncRNA THRIL Is Involved in the Proliferation, Migration, and Invasion of Rheumatoid Fibroblast-like Synoviocytes. Ann. Transl. Med..

[43] Martínez-Ramos S., Rafael-Vidal C., Malvar-Fernández B., Rodriguez-Trillo A., Veale D., Fearon U., Conde C., Conde-Aranda J., Radstake T.R.D.J., Pego-Reigosa J.M. (2023). HOXA5 Is a Key Regulator of Class 3 Semaphorins Expression in the Synovium of Rheumatoid Arthritis Patients. Rheumatology.

[44] Chen Y., Li K., Jiao M., Huang Y., Zhang Z., Xue L., Ju C., Zhang C. (2023). Reprogrammed siTNFα/Neutrophil Cytopharmaceuticals Targeting Inflamed Joints for Rheumatoid Arthritis Therapy. Acta Pharm. Sin. B.

[45] Gilchrist C.L., Francisco A.T., Plopper G.E., Chen J., Setton L.A. (2011). Nucleus Pulposus Cell-Matrix Interactions with Laminins. Eur. Cell Mater..

[46] Banala R.R., Vemuri S.K., Dar G.H., Palanisamy V., Penkulinti M., Surekha M.V., Gurava Reddy A.V., Nalam M.R., Subbaiah G. (2019). Efficiency of Dual siRNA-Mediated Gene Therapy for Intervertebral Disc Degeneration (IVDD). Spine J..

[47] Wang D., Hu Z., Hao J., He B., Gan Q., Zhong X., Zhang X., Shen J., Fang J., Jiang W. (2013). SIRT1 Inhibits Apoptosis of Degenerative Human Disc Nucleus Pulposus Cells through Activation of Akt Pathway. Age.

[48] Lin Y., Yue B., Xiang H., Liu Y., Ma X., Chen B. (2016). Survivin Is Expressed in Degenerated Nucleus Pulposus Cells and Is Involved in Proliferation and the Prevention of Apoptosis In Vitro. Mol. Med. Rep..

[49] Shen J., Fang J., Hao J., Zhong X., Wang D., Ren H., Hu Z. (2016). SIRT1 Inhibits the Catabolic Effect of IL-1β Through TLR2/SIRT1/NF-κB Pathway in Human Degenerative Nucleus Pulposus Cells. Pain Physician.

[50] Bai M., Yin H.-P., Zhao J., Li Y., Wu Y.-M. (2018). Roles of TREM2 in Degeneration of Human Nucleus Pulposus Cells via NF-κB P65. J. Cell. Biochem..

[51] Zhang Z., Xu T., Chen J., Shao Z., Wang K., Yan Y., Wu C., Lin J., Wang H., Gao W. (2021). Correction: Parkin-Mediated Mitophagy as a Potential Therapeutic Target for Intervertebral Disc Degeneration. Cell Death Dis..

[52] Chen J., Zhu H., Xia J., Zhu Y., Xia C., Hu Z., Jin Y., Wang J., He Y., Dai J. (2023). High-Performance Multi-Dynamic Bond Cross-Linked Hydrogel with Spatiotemporal siRNA Delivery for Gene-Cell Combination Therapy of Intervertebral Disc Degeneration. Adv. Sci..

[53] Gibson B.H.Y., Duvernay M.T., Moore-Lotridge S.N., Flick M.J., Schoenecker J.G. (2020). Plasminogen Activation in the Musculoskeletal Acute Phase Response: Injury, Repair, and Disease. Res. Pract. Thromb. Haemost..

[54] Wang L., Yao L., Duan H., Yang F., Lin M., Zhang R., He Z., Ahn J., Fan Y., Qin L. (2021). Plasminogen Regulates Fracture Repair by Promoting the Functions of Periosteal Mesenchymal Progenitors. J. Bone Miner. Res..

[55] Yuasa M., Mignemi N.A., Nyman J.S., Duvall C.L., Schwartz H.S., Okawa A., Yoshii T., Bhattacharjee G., Zhao C., Bible J.E. (2015). Fibrinolysis Is Essential for Fracture Repair and Prevention of Heterotopic Ossification. J. Clin. Investig..

[56] Hang K., Ying L., Bai J., Wang Y., Kuang Z., Xue D., Pan Z. (2021). Knockdown of SERPINB2 Enhances the Osteogenic Differentiation of Human Bone Marrow Mesenchymal Stem Cells via Activation of the Wnt/β-Catenin Signalling Pathway. Stem Cell Res. Ther..

[57] Zhang W., Bai J., Li L., Zhang Y., Hang K., Wang Y., Wang Z., Ye C., Xue D. (2023). EGFL7 Secreted By Human Bone Mesenchymal Stem Cells Promotes Osteoblast Differentiation Partly Via Downregulation Of Notch1-Hes1 Signaling Pathway. Stem Cell Rev. Rep..

[58] Xu X.-M., Xu T.-M., Wei Y.-B., Gao X.-X., Sun J.-C., Wang Y., Kong Q.-J., Shi J.-G. (2018). Low-Intensity Pulsed Ultrasound Treatment Accelerates Angiogenesis by Activating YAP/TAZ in Human Umbilical Vein Endothelial Cells. Ultrasound Med. Biol..

[59] Wang C., Xiao F., Gan Y., Yuan W., Zhai Z., Jin T., Chen X., Zhang X. (2018). Improving Bone Regeneration Using Chordin siRNA Delivered by pH-Responsive and Non-Toxic Polyspermine Imidazole-4,5-Imine. Cell. Physiol. Biochem..

[60] Panupinthu N., Rogers J.T., Zhao L., Solano-Flores L.P., Possmayer F., Sims S.M., Dixon S.J. (2008). P2X7 Receptors on Osteoblasts Couple to Production of Lysophosphatidic Acid: A Signaling Axis Promoting Osteogenesis. J. Cell Biol..

[61] Bara T., Synder M., Studniarek M. (2000). The Application of Shock Waves in the Treatment of Delayed Bone Union and Pseudoarthrosis in Long Bones. Ortop. Traumatol. Rehabil..

[62] Sun D., Junger W.G., Yuan C., Zhang W., Bao Y., Qin D., Wang C., Tan L., Qi B., Zhu D. (2013). Shockwaves Induce Osteogenic Differentiation of Human Mesenchymal Stem Cells through ATP Release and Activation of P2X7 Receptors. Stem Cells.

[63] Liu X. (2016). Bone Site-Specific Delivery of siRNA. J. Biomed. Res..

[64] Bologna J.-C., Dorn G., Natt F., Weiler J. (2003). Linear Polyethylenimine as a Tool for Comparative Studies of Antisense and Short Double-Stranded RNA Oligonucleotides. Nucleosides Nucleotides Nucleic Acids.

[65] Qadir A., Gao Y., Suryaji P., Tian Y., Lin X., Dang K., Jiang S., Li Y., Miao Z., Qian A. (2019). Non-Viral Delivery System and Targeted Bone Disease Therapy. Int. J. Mol. Sci..

[66] Conte R., Finicelli M., Borrone A., Margarucci S., Peluso G., Calarco A., Bosetti M. (2023). MMP-2 Silencing through siRNA Loaded Positively-Charged Nanoparticles (AcPEI-NPs) Counteracts Chondrocyte De-Differentiation. Polymers.

[67] Wang Y., Tran K.K., Shen H., Grainger D.W. (2012). Selective Local Delivery of RANK siRNA to Bone Phagocytes Using Bone Augmentation Biomaterials. Biomaterials.

[68] Moldovan F. (2023). Bone Cement Implantation Syndrome: A Rare Disaster Following Cemented Hip Arthroplasties-Clinical Considerations Supported by Case Studies. J. Pers. Med..

[69] Peng L., Wagner E. (2019). Polymeric Carriers for Nucleic Acid Delivery: Current Designs and Future Directions. Biomacromolecules.

[70] Al-Absi M.Y., Caprifico A.E., Calabrese G. (2023). Chitosan and Its Structural Modifications for siRNA Delivery. Adv. Pharm. Bull..

[71] Wang F., Pang J.-D., Huang L.-L., Wang R., Li D., Sun K., Wang L.-T., Zhang L.-M. (2018). Nanoscale Polysaccharide Derivative as an AEG-1 siRNA Carrier for Effective Osteosarcoma Therapy. Int. J. Nanomed..

[72] Minakuchi Y., Takeshita F., Kosaka N., Sasaki H., Yamamoto Y., Kouno M., Honma K., Nagahara S., Hanai K., Sano A. (2004). Atelocollagen-Mediated Synthetic Small Interfering RNA Delivery for Effective Gene Silencing in Vitro and in Vivo. Nucleic Acids Res..

[73] Takeshita F., Minakuchi Y., Nagahara S., Honma K., Sasaki H., Hirai K., Teratani T., Namatame N., Yamamoto Y., Hanai K. (2005). Efficient Delivery of Small Interfering RNA to Bone-Metastatic Tumors by Using Atelocollagen in Vivo. Proc. Natl. Acad. Sci. USA.

[74] Srivastava A., Prajapati A. (2020). Albumin and Functionalized Albumin Nanoparticles: Production Strategies, Characterization, and Target Indications. Asian Biomed..

[75] Chen J., Wu Z., Ding W., Xiao C., Zhang Y., Gao S., Gao Y., Cai W. (2020). SREBP1 siRNA Enhance the Docetaxel Effect Based on a Bone-Cancer Dual-Targeting Biomimetic Nanosystem against Bone Metastatic Castration-Resistant Prostate Cancer. Theranostics.

[76] Ito M., Yurube T., Kakutani K., Maeno K., Takada T., Terashima Y., Kakiuchi Y., Takeoka Y., Miyazaki S., Kuroda R. (2017). Selective interference of mTORC1/RAPTOR protects against human disc cellular apoptosis, senescence, and extracellular matrix catabolism with Akt and autophagy induction. Osteoarthr. Cartil..

[77] Aldayel A.M., O’Mary H.L., Valdes S.A., Li X., Thakkar S.G., Mustafa B.E., Cui Z. (2018). Lipid nanoparticles with minimum burst release of TNF-α siRNA show strong activity against rheumatoid arthritis unresponsive to methotrexate. J. Control. Release.

[78] Cui Y., Guo Y., Kong L., Shi J., Liu P., Li R., Geng Y., Gao W., Zhang Z., Fu D. (2022). A bone-targeted engineered exosome platform delivering siRNA to treat osteoporosis. Bioact. Mater..

[79] Song W., Song X., Yang C., Gao S., Klausen L.H., Zhang Y., Dong M., Kjems J. (2015). Chitosan/siRNA functionalized titanium surface via a layer-by-layer approach for in vitro sustained gene silencing and osteogenic promotion. Int. J. Nanomed..

[80] Kang M.A., Rao P.P., Matsui H., Mahajan S.S. (2022). Delivery of mGluR5 siRNAs by Iron Oxide Nanocages by Alternating Magnetic Fields for Blocking Proliferation of Metastatic Osteosarcoma Cells. Int. J. Mol. Sci..

[81] Zhang Y., Wei L., Miron R.J., Shi B., Bian Z. (2015). Anabolic Bone Formation via a Site-Specific Bone-Targeting Delivery System by Interfering with Semaphorin 4D Expression. J. Bone Miner. Res..

[82] Zheng G., Peng X., Zhang Y., Wang P., Xie Z., Li J., Liu W., Ye G., Lin Y., Li G. (2023). A Novel Anti-ROS Osteoblast-Specific Delivery System for Ankylosing Spondylitis Treatment via Suppression of Both Inflammation and Pathological New Bone Formation. J. Nanobiotechnol..

[83] Bartok B., Hammaker D., Firestein G.S. (2014). Phosphoinositide 3-Kinase δ Regulates Migration and Invasion of Synoviocytes in Rheumatoid Arthritis. J. Immunol..

[84] Li J., Lee W.Y., Wu T., Xu J., Zhang K., Li G., Xia J., Bian L. (2016). Multifunctional Quantum Dot Nanoparticles for Effective Differentiation and Long-Term Tracking of Human Mesenchymal Stem Cells In Vitro and In Vivo. Adv. Healthc. Mater..

[85] Liu J., Jiang T., Li C., Wu Y., He M., Zhao J., Zheng L., Zhang X. (2019). Bioconjugated Carbon Dots for Delivery of siTnfα to Enhance Chondrogenesis of Mesenchymal Stem Cells by Suppression of Inflammation. Stem Cells Transl. Med..

[86] Zhang S., Zhi D., Huang L. (2012). Lipid-Based Vectors for siRNA Delivery. J. Drug Target..

[87] Sørensen D.R., Leirdal M., Sioud M. (2003). Gene Silencing by Systemic Delivery of Synthetic siRNAs in Adult Mice. J. Mol. Biol..

[88] Bose A., Roy Burman D., Sikdar B., Patra P. (2021). Nanomicelles: Types, Properties and Applications in Drug Delivery. IET Nanobiotechnol..

[89] Nsairat H., Khater D., Sayed U., Odeh F., Al Bawab A., Alshaer W. (2022). Liposomes: Structure, Composition, Types, and Clinical Applications. Heliyon.

[90] Barenholz Y. (2012). Doxil^®^—the First FDA-Approved Nano-Drug: Lessons Learned. J. Control. Release.

[91] Jeong G.H., Nam M.K., Hur W., Heo S., Lee S., Choi E., Park J.H., Park Y., Kim W.U., Rhim H. (2023). Role of High-Temperature Requirement Serine Protease A 2 in Rheumatoid Inflammation. Arthritis Res. Ther..

[92] Stapleton M., Sawamoto K., Alméciga-Díaz C.J., Mackenzie W.G., Mason R.W., Orii T., Tomatsu S. (2017). Development of Bone Targeting Drugs. Int. J. Mol. Sci..

[93] Ma X., Gao Y., Zhao D., Zhang W., Zhao W., Wu M., Cui Y., Li Q., Zhang Z., Ma C. (2021). Titanium Implants and Local Drug Delivery Systems Become Mutual Promoters in Orthopedic Clinics. Nanomaterials.

[94] Vallabani N.V.S., Singh S. (2018). Recent Advances and Future Prospects of Iron Oxide Nanoparticles in Biomedicine and Diagnostics. 3 Biotech.

[95] Kang M.A., Fang J., Paragodaarachchi A., Kodama K., Yakobashvili D., Ichiyanagi Y., Matsui H. (2022). Magnetically Induced Brownian Motion of Iron Oxide Nanocages in Alternating Magnetic Fields and Their Application for Efficient siRNA Delivery. Nano Lett..

[96] Singh K.R., Nayak V., Sarkar T., Singh R.P. (2020). Cerium Oxide Nanoparticles: Properties, Biosynthesis and Biomedical Application. RSC Adv..

[97] Betty C.A. (2008). Porous Silicon: A Resourceful Material for Nanotechnology. Recent Pat. Nanotechnol..

[98] Obonyo O., Fisher E., Edwards M., Douroumis D. (2010). Quantum Dots Synthesis and Biological Applications as Imaging and Drug Delivery Systems. Crit. Rev. Biotechnol..

[99] Yukawa H., Sato K., Baba Y. (2023). Theranostics Applications of Quantum Dots in Regenerative Medicine, Cancer Medicine, and Infectious Diseases. Adv. Drug Deliv. Rev..

[100] San Juan A., Bala M., Hlawaty H., Portes P., Vranckx R., Feldman L.J., Letourneur D. (2009). Development of a Functionalized Polymer for Stent Coating in the Arterial Delivery of Small Interfering RNA. Biomacromolecules.

[101] Zhong R., Talebian S., Mendes B.B., Wallace G., Langer R., Conde J., Shi J. (2023). Hydrogels for RNA Delivery. Nat. Mater..

[102] Park J.S., Yang H.N., Jeon S.Y., Woo D.G., Kim M.S., Park K.-H. (2012). The Use of Anti-COX2 siRNA Coated onto PLGA Nanoparticles Loading Dexamethasone in the Treatment of Rheumatoid Arthritis. Biomaterials.

[103] Ahn I., Kang C.S., Han J. (2023). Where Should siRNAs Go: Applicable Organs for siRNA Drugs. Exp. Mol. Med..

[104] De Vivo M., Dal Peraro M., Klein M.L. (2008). Phosphodiester Cleavage in Ribonuclease H Occurs via an Associative Two-Metal-Aided Catalytic Mechanism. J. Am. Chem. Soc..

[105] Imaeda A., Tomoike F., Hayakawa M., Nakamoto K., Kimura Y., Abe N., Abe H. (2019). N6-Methyl Adenosine in siRNA Evades Immune Response without Reducing RNAi Activity. Nucleosides Nucleotides Nucleic Acids.

[106] Meng Z., Lu M. (2017). RNA Interference-Induced Innate Immunity, Off-Target Effect, or Immune Adjuvant?. Front. Immunol..

[107] Liu J., Brutkiewicz R.R. (2017). The Toll-like receptor 9 signalling pathway regulates MR 1-mediated bacterial antigen presentation in B cells. Immunology.

[108] Tai W. (2019). Current Aspects of siRNA Bioconjugate for In Vitro and In Vivo Delivery. Molecules.

[109] Kittler R., Surendranath V., Heninger A.-K., Slabicki M., Theis M., Putz G., Franke K., Caldarelli A., Grabner H., Kozak K. (2007). Genome-Wide Resources of Endoribonuclease-Prepared Short Interfering RNAs for Specific Loss-of-Function Studies. Nat. Methods.

[110] Ambike S., Cheng C.-C., Feuerherd M., Velkov S., Baldassi D., Afridi S.Q., Porras-Gonzalez D., Wei X., Hagen P., Kneidinger N. (2022). Targeting Genomic SARS-CoV-2 RNA with siRNAs Allows Efficient Inhibition of Viral Replication and Spread. Nucleic Acids Res..

